# Novel biomarkers derived from the Maintenance of Wakefulness Test as predictors of sleepiness and response to treatment

**DOI:** 10.1093/sleep/zsae148

**Published:** 2024-07-02

**Authors:** Brian Tracey, Mark Culp, Stephan Fabregas, Emmanuel Mignot, Derek L Buhl, Dmitri Volfson

**Affiliations:** Statistical and Quantitative Sciences, Takeda Development Center Americas, Inc., Cambridge, MA, USA; Stat Tenacity, LLC, Saline, MI, USA; Signal Insights, LLC, Cambridge, MA, USA; Stanford Department of Psychiatry and Behavioral Sciences, Center for Sleep Sciences and Medicine, Stanford University Medical School, Palo Alto, CA, USA; Statistical and Quantitative Sciences, Takeda Development Center Americas, Inc., Cambridge, MA, USA; Statistical and Quantitative Sciences, Takeda Development Center Americas, Inc., Cambridge, MA, USA

**Keywords:** maintenance of wakefulness, MWT, excessive daytime sleepiness, sleep onset latency, narcolepsy, TAK-925, danavorexton, orexin, qEEG, hypnodensity

## Abstract

The Maintenance of Wakefulness Test (MWT) is a widely accepted objective test used to evaluate daytime somnolence and is commonly used in clinical studies evaluating novel therapeutics for excessive daytime sleepiness. In the latter, sleep onset latency (SOL) is typically the sole MWT endpoint. Here, we explored microsleeps, sleep probability measures derived from automated sleep scoring, and quantitative electroencephalography (qEEG) features as additional MWT biomarkers of daytime sleepiness, using data from a phase 1B trial of the selective orexin receptor 2 agonist danavorexton (TAK-925) in people with narcolepsy type 1 (NT1) or type 2 (NT2). Danavorexton treatment reduced the rate and duration of microsleeps during the MWT in NT1 (days 1 and 7; *p *≤ .005) and microsleep rate in NT2 (days 1 and 7; *p *< .0001). The use of an EEG-sleep-staging − derived measure to determine the probability of wakefulness for each minute revealed a novel metric to track changes in daytime sleepiness, which were consistent with the θ/α ratio, a known biomarker of drowsiness. The slopes of line-fits to both the log-transformed sleepiness score or log-transformed θ/α ratio correlated well to (inverse) MWT SOL for NT1 (R = 0.93 and R = 0.83, respectively) and NT2 (R = 0.97 and R = 0.84, respectively), suggesting that individuals with narcolepsy have increased sleepiness immediately after lights-off. These analyses demonstrate that novel EEG-based biomarkers can augment SOL as predictors of sleepiness and its response to treatment and provide a novel framework for the analysis of wake EEG in hypersomnia disorders.

## Graphical Abstract

**Graphical Abstract:**
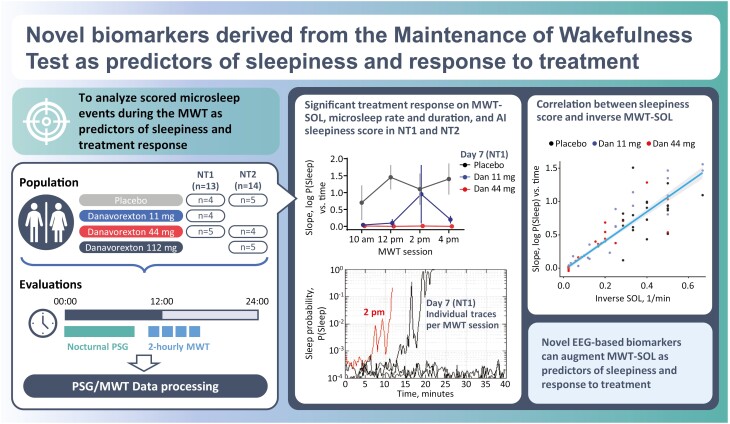

**Statement of Significance**

Electroencephalography (EEG) data contained in Maintenance of Wakefulness Test (MWT) recordings are typically underutilized but may provide useful insights into daytime wakefulness. We demonstrate that evaluation of microsleeps and other EEG-derived metrics during the MWT can reveal statistically significant treatment effects and track changes in daytime sleepiness in people with narcolepsy types 1 and 2. Participants receiving treatment with the orexin 2 receptor agonist danavorexton had the expected delayed onset to sustained sleep but also had fewer microsleep episodes prior to sleep onset. In addition, both artificial intelligence−derived and standard quantitative EEG biomarkers are sensitive to treatment effects and may help predict sleep onset latency. These analyses provide a novel framework for the analysis of wake EEG in hypersomnia disorders.

People living with central disorders of hypersomnolence, including narcolepsy type 1 (NT1), narcolepsy type 2 (NT2), and idiopathic hypersomnia, typically report excessive daytime sleepiness (EDS) as being their most debilitating symptom, with significant impairment of functioning and everyday activities [1–3]. Subjective measures that rely on individual recall such as the Epworth Sleepiness Scale are the only tools available to evaluate EDS outside of the clinic environment [4]. In the clinical setting, the Maintenance of Wakefulness Test (MWT) [5] is used to objectively assess an individual’s capacity to remain awake while sitting quietly in a dark room. Together with the Epworth Sleepiness Scale, it is the accepted approach to evaluate novel wake-promoting pharmacology [6, 7]. In addition, the MWT offers an opportunity to assess an individual’s ability to maintain alertness and is occasionally used to assess whether a treated patient is sufficiently alert, although more normative data and real-world assessments are needed [7].

MWTs are typically performed four times daily at 2-hour intervals to capture fluctuations in sleepiness throughout the day. Each MWT session consists of a 40-minute wake trial, with the initial trial beginning 90–180 minutes after the individual wakes up from the previous night’s sleep [8]. Electroencephalography (EEG), electromyography (EMG), and electro-oculography (EOG) are used for the assessment of sleep onset latency (SOL) by a sleep technician, the primary outcome of interest. Mean SOL across the four MWT episodes during the day is typically calculated, although measurement of SOL for each individual episode may provide more detailed information. In addition to the evaluation of people with EDS, MWT SOL has been studied in healthy populations [9, 10] and has been shown to correlate with poor driving performance [11–14]. Although MWT SOL is widely accepted as an objective measure of alertness, EEG data gathered during the MWT is often underutilized, even though the rich dataset may contain additional information relating to daytime sleepiness. Specifically, useful new biomarkers could be extracted from the EEG data that highlight different aspects of daytime sleepiness. Indeed, the MWT SOL reveals whether individuals are capable of delaying persistent sleep but does not reveal whether individuals are awake but drowsy (having difficulty fighting off sleep and perhaps dipping into brief periods of sleep, or “microsleeps”) or whether they are truly awake and alert (i.e., far from sleep during the test) [7]. The MWT SOL is also highly modulated by the individual’s level of motivation for performing well in the test, thus affecting the outcome [7].

One area of active research to leverage the rich data contained in MWT recordings is the analysis of microsleep episodes (MSEs), defined here as sleep episodes ≥3 to <15 seconds in length [15]. Quantitative EEG (qEEG) biomarkers of daytime sleepiness can also be explored using EEGs obtained during the MWT. Subjective sleepiness in the EEG has been shown to be negatively correlated with α band power (8–12 Hz) at all locations and positively correlated with θ power (4–8 Hz) in frontal locations [16]. Cantero et al. found that during drowsiness there was an increase in EEG power in occipital channels in the 9.5–11 Hz range [17]. Similarly, Kim et al. [18] and Melia et al. [19] showed that sleep onset is associated with changes in α activity and θ/α ratio. Beyond standard qEEG analysis, recent work has seen the development of deep learning methods that combine multiple channels to produce estimates of sleep state probability [20]. Here, we expand on these efforts and identify novel biomarkers of daytime sleepiness relevant to NT1 and NT2 using MWT data obtained from a clinical trial of danavorexton (TAK-925), a selective orexin receptor 2 agonist (NCT03748979).

## Methods

### Population and data collection

Data from a phase 1B trial of the selective orexin receptor 2 agonist (OX2R) danavorexton (TAK-925) in people with NT1 (*n* = 13) or NT2 (*n* = 14) were evaluated. Participants with NT1 were randomized to placebo (denoted Bp, *n* = 4), 11 mg danavorexton (B1, *n* = 4), and 44 mg danavorexton (B2, *n* = 5). Participants with NT2 were randomized to placebo (Cp, *n* = 5), 44 mg danavorexton (C1, *n* = 4), and 112 mg danavorexton (C2, *n* = 5). Lower doses were tested in the NT1 population, as it was expected that the orexin agonist would have higher efficacy in a population lacking orexin at baseline [21].

All MWT sessions were conducted following a night of nocturnal polysomnography (PSG). Participants were not allowed to consume caffeine prior to the MWTs. Following baseline PSG and MWTs, participants received daily 9-hour infusions of either placebo or danavorexton for seven consecutive days. Patients were asked to rate their sleepiness every 2 hours starting at 9:00 am using the Karolinska Sleepiness Scale (KSS) (9-point scale, with 1 being extremely alert and 9 being extremely sleepy). To assess wakefulness, standard 40-minute/4-trial MWT sessions were conducted at 10:00 am, 12:00 pm, 2:00 pm, and 4:00 pm, with the standard 2-hour gap between the start of each session [8]. Participants were required to stay awake between the sessions. MWTs were performed the day before drug administration (baseline), as well as on days 1 and 7 of the infusion periods.

### Data processing methods and variable definitions

#### Manual scoring of SOL and microsleeps

MWT SOL was determined by trained technicians based on standard manual sleep stage scoring rules (30-second epoch length) of EEG/EOG/EMG recordings [22]. SOL was defined from unambiguous sleep onset, taken as three consecutive epochs of stage N1 or a single epoch of any deeper sleep stage, i.e. N2, N3, or REM (note that SOL may alternatively be defined as time to the first epoch of any sleep stage [7]). EEG consisted of frontal (F3, F4), central (C3, C4), and occipital (O1, O2) channels, each referenced to its contralateral mastoid. EOG consisted of left and right outer canthus referenced to the right mastoid. EMG consisted of three mandible leads, two of which were selected as reference derivation, while the third was used as a backup in the event of data quality issues with the primary leads.

Microsleep scoring was performed by an expert sleep scorer who was fully blinded (to the participant, MWT session, day and treatment group, lights-off times, and previous sleep stage scoring completed by other trained technicians). EEG/EOG/EMG/electrocardiogram recordings with the montage described above were scored; this included data from approximately 5 minutes before lights off to 5 minutes after lights on. The scorer was not provided any information about lights-off/on times or previous scoring on 30-second epochs, but in this analysis, only microsleeps after lights-off and before lights-on were considered. Microsleeps, defined as sleep periods lasting between 3 and 15 seconds, were scored based on visual inspection of EEG channels (frontal, central, and occipital) as well as EOG and EMG. We defined a microsleep as a slowing in the EEG (shift from α to θ), with dominant θ (4–8 Hz) activity, often accompanied by slow rolling eye movements and occasionally accompanied by sleep spindles and/or K complexes (see Supplementary Figure S1 for an example). The start time and duration of each microsleep were noted and compiled for later analysis.

Scored microsleeps were used to compute the following metrics: (1) microsleep rate, defined as the number of microsleeps per 30-second epoch during a session, and (2) microsleep duration, defined as the average length of a microsleep in a session. If no microsleeps were detected during a session, the microsleep rate and microsleep duration were reported as 0.

### Sleepiness score derived from automated sleep scoring

Automated sleep scoring was performed using the approach described in Stephansen et al. [20] (code available at https://github.com/Stanford-STAGES). Note that this code consists of two separate steps. First, sleep “hypnodensities” are created for 15-second epochs, giving the probability of the participant being in each of the five standard sleep states. Secondly, features derived from these hypnodensities are used to analyze whole-night PSG for detecting NT1. In this work, we only conducted the first stage of processing (hypnodensity sleep stage probability generation per epoch), ignoring the NT1 classification stage.

Following the estimation of hypnodensities for the MWT, we derived an overall “sleepiness score” at the 15-second time resolution by summing probabilities of all non-wake sleep states. Because this algorithm operates at a 15-second timescale, which is insufficient for detecting microsleeps, we did not attempt to use it as a tool for microsleep detection but instead elected to explore changes in wakefulness during the MWT.

Several summary metrics were defined to capture a sleepiness score in each session. First, the average sleepiness score across the entire session was computed. Linear regressions were then fit to log-transformed sleepiness scores across the session; the resulting slope and intercept defined the “sleepiness score slope” and “sleepiness score intercept” metrics for each session. The sleepiness intercept quantified how sleepy participants were at the beginning of the MWT session, while the slope captured the increase in sleepiness observed during the session until sleep onset.

### qEEG analysis

qEEG results were generated for an occipital channel (O2) referenced to the contralateral mastoid. Before computing qEEG metrics (such as the theta–alpha metric shown below), EEG channels were band-pass filtered at 0.1–55.0 Hz, and notch filters with a bandwidth of 1 were applied at specific frequencies across recordings for which the spectra for that record showed noise in that frequency well above the floor (for example, if there was harmonic noise from line interference). These notch filters were selected based on visual inspection. No filtering (beyond that applied in the sleep lab) was applied to data used for microsleep scoring or AI-based sleepiness score computation.

Although Kim et al. [18] used the detrended fluctuation analysis (DFA) heuristic to analyze sleep onset, recent work has shown that DFA does not handle non-stationarity well [23], so we instead use more standard time-frequency analysis. Spectrum estimates were generated every 2 seconds at 0.5 Hz resolution using multi-taper methods [24], and averaged down to 15-second epochs after automated rejection of time windows with detected data quality problems (suspected artifacts, missing samples, high slew rate, and saturation). The θ/α ratio was computed as a marker of drowsiness, with θ band defined as 4–8 Hz and α defined as 8–12 Hz.

As with the sleepiness score, the θ/α ratio in each session was captured using an average θ/α ratio. Then, a linear regression was fit to log-transformed θ/α ratio across the session. The resulting slope and intercept were tabulated, defining the “θ/α slope” and “θ/α intercept” metrics for each session.

### Statistical analysis

Modeling endpoints for this analysis included SOL, microsleep rate, microsleep duration, sleepiness score, sleepiness score slope, sleepiness score intercept, θ/α ratio, θ/α slope, θ/α intercept, and KSS score (with measurements before and after each MWT session linearly interpolated to match the MWT timepoints). Linear mixed-effects models were used to analyze the change from baseline in each cohort for each endpoint, as well as for assessing correlations over time [25].

An extensive model selection process was performed using microsleep rate as the primary microsleep measure for analysis. This model selection process, based on the penalized log-likelihood metric referred to as the Akaike information criterion (AIC) [26], explored the effect of MWT session and showed that the 02:00 pm session behaved differently from other sessions, most likely reflecting the “siesta effect,” or increased sleepiness that typically occurs in the early afternoon [27]. Model selection led to a simplified model that separated the 02:00 pm session from the other three sessions. Thus, an indicator variable “isSiesta” was used to denote the 02:00 pm session in the data. Then, each endpoint was analyzed using day, treatment, isSiesta, and the interaction of day and treatment as fixed effects, and participant as a random effect (i.e. random intercept per participant). To allow comparison of models from different endpoints, the model above (selected based on microsleep rate analysis) was applied to all endpoints.

Statistical analyses were performed using R version 4.2.2. Repeated measures correlations were computed using the “rmcorr” package. For EEG-derived metrics (sleepiness score and θ/α metrics) the “lme4” package with the lmer function was used to fit models. Of note, this package had convergence errors for the microsleep rate metric and microsleep duration metric. Specifically, day 1 treatment B2 did not converge due to zero microsleep counts for every participant in the group, which caused standard models to fail (i.e. estimates and standard errors tend to infinity). It is worth noting that this convergence issue is a sign of efficacy (i.e. it was due to the fact the participants were entirely awake).

#### Bayesian modeling approach

To manage convergence issues, a Bayesian approach (“brms” package), capable of handling microsleep data in which counts may go to zero, was adopted. In this Bayesian approach, a normal prior was applied to the fixed-effect coefficient that stabilizes the estimates, that is, the model was fit with the additional assumption that the fixed-effect coefficient is a N(0,t) random variable with hyper-parameter *t* > 0 (this is not applied to the intercept). Selection of the hyper-parameter t involves a bias/variance tradeoff. As t gets smaller the estimates tend to zero, that is, the signal is not able to detect effects (low variance, high bias). As t increases in magnitude, the model becomes more data-driven and less stable (high variance, low bias). To mitigate these effects, we tested a range of values for t and computed two commonly used performance metrics, the Watanabe–AIC and leave-one-out cross-validation (LOO). These measures optimized an error rate and lead to the best overall model. An inflection point at *t* = 2.963 was clearly observed and this value was used for the hyper-parameter t in subsequent modeling of microsleep events.

## Results

### MWT SOL

**Figure 1.**
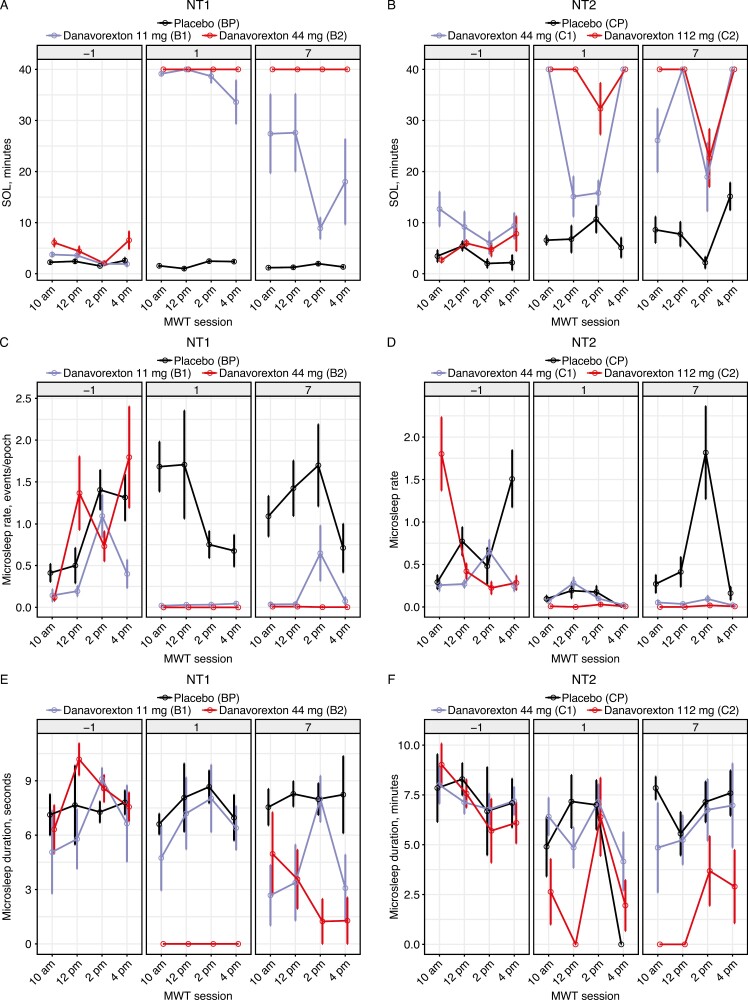
Maintenance of Wakefulness Test (MWT) sleep onset latency (SOL) and microsleep metrics by treatment group and day for participants with narcolepsy type 1 (NT1) (A, C, E) and narcolepsy type 2 (NT2; B, D, F). SOL (A, B) shows a clear treatment effect. A siesta effect was apparent at 02:00 pm in NT1 and NT2, also visible without treatment. Microsleep rate (C, D) decreases greatly during treatment, with microsleeps basically eliminated on day 1. Microsleep duration (E, F) is also reduced in the NT1 cohort. Cohort values: Bp, T1 placebo; B1, NT1 11 mg danavorexton; B2, NT1 44 mg danavorexton; Cp, NT2 placebo; C1, NT2 44 mg danavorexton; C2, NT2 112 mg danavorexton. One epoch, 30 seconds.

Administration of danavorexton significantly increased MWT SOL in both the NT1 and NT2 cohorts (Figure 1, A and B). All participants stayed awake for >20 minutes in each session and almost all participants with NT1 stayed awake throughout all four of the 40-minute MWT sessions. Computed contrasts from mixed-model analysis of MWT SOL are shown in Supplementary Tables S1 and S2. A siesta effect (shorter sleep latency at 02:00 pm) was evident at the lower doses for each cohort. Of note, the danavorexton infusion protocol was designed to maintain stable concentrations all through the day; thus, this siesta effect represents the appearance of a well-characterized human MWT/Multiple Sleep Latency Test feature found in normal untreated participants [27], which was unmasked by the therapy.

Administration of danavorexton also significantly reduced KSS scores of self-reported sleepiness (Supplementary Tables S1 and S2). KSS reductions were noticeably larger in the NT1 cohorts (3.3- or 4.1-point reductions at day 7) than in NT2 (2- or 1.3-point reduction at day 7).

### Microsleep analysis

Microsleep rate and duration were significantly reduced from baseline with danavorexton treatment in the NT1 and NT2 cohorts (Figure 1C–F). Thus, microsleeps were frequently observed at baseline (in 40% of NT1 MWT trials and 48% of NT2 trials) but were less frequently observed in the overall dataset (in 29% of NT1 trials and 34% of NT2 trials, across all 3 days). Baseline-adjusted microsleep rate and duration were significantly reduced in the NT1 cohorts on treatment days 1 and 7 (Figure 1C, E; mixed-model contrasts, *p* < .005 vs. baseline with the 11 mg dose, *p* ≤ .0001 vs. baseline with the 44 mg dose; Supplementary Table S1). No significant changes were observed in the NT1 placebo group on either day. For the NT2 cohorts, microsleep rate and duration decreased significantly from baseline after treatment with the 112 mg dose of danavorexton (Figure 1D, F; mixed-model contrasts *p* < .0001 for both days; Supplementary Table S2), and with 44 mg danavorexton on day 1 (rate, *p* < .0001; duration, *p* = .0136) and day 7 (*p* < .0001 for both). Significant decreases from baseline were also observed in the NT2 placebo group on day 1 (rate, *p* = .004; duration, *p* < .001; Supplementary Table S2). The siesta effect was pronounced for both microsleep rate and duration in both the NT1 cohort (*p* = .047 for both) and in the NT2 cohort (*p* = .007 and *p* = .014, respectively).

### Sleep probability and qEEG analysis

**Figure 2.**
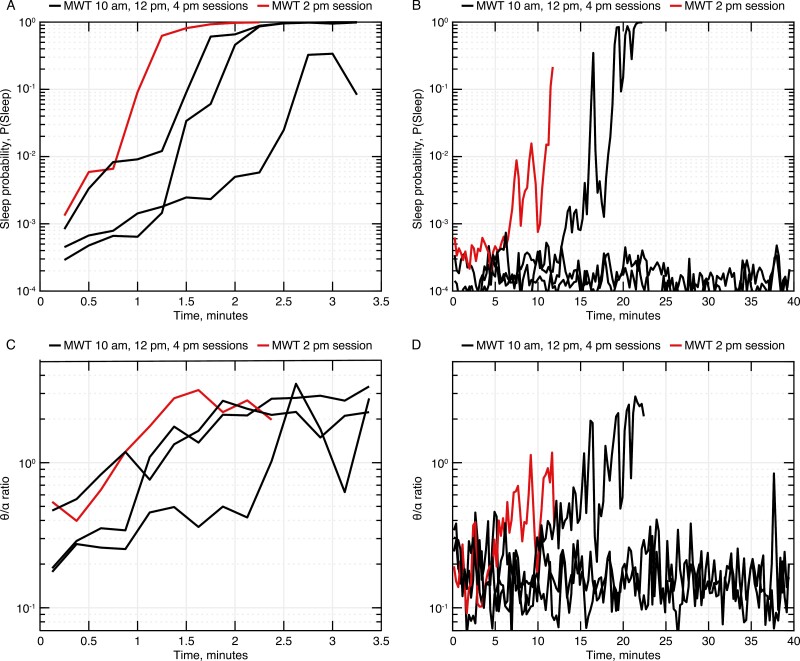
Log-transformed sleepiness scores (A, B) and θ/α ratios (C, D) for an example participant with narcolepsy type 1 (cohort B1), for all 4 MWT sessions on the baseline visit day (A, C) and on treatment day 7 (B, D). Each line shows the response versus time during a single Maintenance of Wakefulness Test session. Note that treatment increases the time subjects remain awake, so the time duration on day 7 is much longer. Also, note the general consistency between sleepiness scores and θ/α ratio values.

For the NT1 cohorts, the θ/α ratio curves from qEEG traces were generally consistent with sleepiness scores (Figure 2; example for one individual with NT1 at baseline and day 7), though sleepiness scores appear less “noisy” than θ/α ratios. An upward trajectory starting at lights-off on untreated days suggests increasing sleepiness as soon as lights are turned off in participants with NT1. On the treatment day, several sessions indicated no increase after lights-off, suggesting that wakefulness is maintained. During the 02:00 pm session (Figure 2; highlighted in red), a more rapid increase in sleepiness was observed, typical for many participants, consistent with the siesta effect observed in the MWT SOL. Computed contrasts from mixed-model analysis of average, slope, and intercept metrics for both the sleepiness score and θ/a ratio are shown in Supplementary Tables S1 and S2.

**Figure 3.**
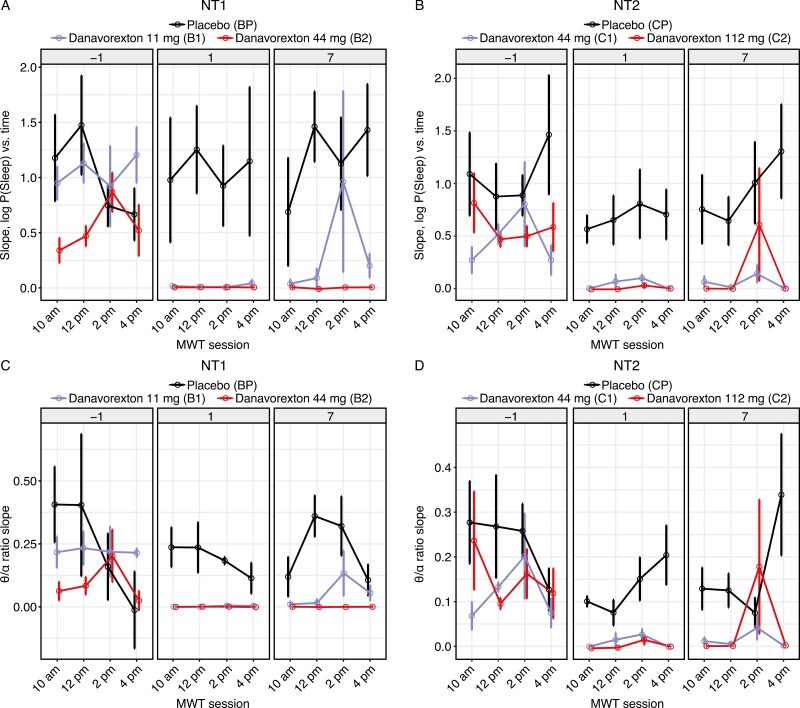
Changes in electroencephalogram-derived Maintenance of Wakefulness Test endpoints, by treatment group and day, for participants with narcolepsy type 1 (NT1) (A, C) and narcolepsy type 2 (NT2; B, D): (A, B) slopes of sleepiness scores and (C, D) θ/α ratios. Cohort values: Bp, NT1 placebo; B1, NT1 11 mg danavorexton; B2, NT1 44 mg danavorexton; Cp, NT2 placebo; C1, NT2 44 mg danavorexton; C2, NT2 112 mg danavorexton.

Cohort-level “sleepiness” scores demonstrate that all three cohorts exhibited similar upward slopes at baseline, which were substantially reduced during treatment at all tested danavorexton doses in participants with either NT1 or NT2 (Figure 3, A and B, respectively). The changes in the “sleepiness” scores were consistent with those observed in the θ/α ratio (Figure 3, C and D). This reduction in slope was strongest on day 1 (acute effect) but was clearly seen on day seven after a week of continuous dosing. A tendency for increased slopes during the 02:00 pm MWT session was observed, illustrating the siesta effect.

Although slopes were reduced by treatment, the intercept metric was not (Supplementary Tables S1 and S2). These data suggest that both treated and untreated participants (in general) started the MWT session at a comparable level of sleepiness but that the rate of increase in sleepiness was much lower in treated participants.

### Correlation of EEG-derived and microsleep metrics with MWT SOL and KSS

**Figure 4.**
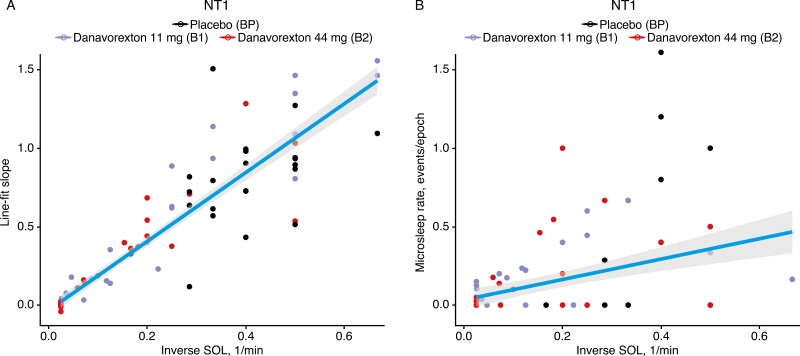
Correlations between (A) sleepiness slope score and (B) microsleep rate with 1/(Maintenance of Wakefulness Test sleep onset latency [SOL]) for the narcolepsy type 1 (NT1) cohort. Each dot represents an individual participant/MWT session (note that analysis was limited to sessions in which the SOL was > 1 minute in order to improve the reliability of slope estimates). Cohort values: Bp, NT1 placebo; B1, NT1 11 mg danavorexton; B2, NT1 44 mg danavorexton. Regression lines are for visual guidance; correlations accounting for repeated measures within subjects are reported in Supplementary Tables S3 and S4.

We next evaluated correlations between EEG-derived endpoints and microsleep metrics with MWT SOL times, limiting analysis to sessions in which the SOL was >1 minute in order to improve the reliability of slope estimates. For NT1, the sleepiness score slopes were well correlated with inverse SOL (1/MWT SOL); thus, lower slopes correspond to later SOLs (Figure 4A). Repeated measures correlation coefficients between inverse SOL and the sleepiness and θ/α metrics described in the previous section are shown in Supplementary Table S3. For NT1, a high correlation between inverse MWT SOL and sleepiness slope score was observed (R = 0.90, *p* < 1e–5), with a slightly lower correlation between inverse MWT SOL and θ/α slope (R = 0.83, *p* < 1e–5). Moderate correlations were observed for average sleepiness during the session (R = 0.45, *p* < 1e–5). Similar results were seen for NT2: an inverse MWT SOL was highly correlated with sleepiness score slope (R = 0.95, *p* < 1e–5) and the θ/α slope (R = 0.84, *p* < 1e–5), with a moderate correlation with average sleepiness score (R = 0.50, *p* < 1e–5).

Supplementary Table S3 also shows correlations between qEEG-derived metrics, the sleepiness slope, and the θ/α slope. Consistent with the sleepiness scores (Figure 3), these metrics are well correlated for both NT1 (R = 0.90, *p* < 1e–5) and NT2 (R = 0.86, *p* < 1–5).

We found that the inverse MWT SOL had low-to-moderate (though still significant) correlations to microsleep rate (Figure 4B; correlation for NT1 was R = 0.26, *p* < 1e–2; for NT2, R = 0.42, *p* < 1e–5; Supplementary Table S3). This lower correlation was seen because most sessions did not have any microsleeps; limiting the ability to predict SOL based on microsleep data. For participants who did experience microsleeps, Figure 4B shows the rate increased as MWT SOL decreased (i.e. as 1/MWT SOL increases). The correlations between microsleep metrics with EEG-derived metrics were weak or insignificant (see Supplementary Table S4).

Correlations of other endpoints versus KSS are listed in Supplementary Table S5. While several endpoints show a significant correlation to KSS, the degree of correlation to KSS was generally lower than was observed among the objective endpoints, particularly in NT2 subjects. The highest correlations were observed for sleepiness slope (correlation R = 0.63, *p* < 1e–5 in NT1; R = 0.36, *p* < 1e–5 in NT2) and inverse MWT SOL (R = 0.64, *p* < 1e–5 in NT1; R = 0.42, *p* < 1e–5 in NT2).

## Discussion

MWT SOL is commonly used as a clinical indicator of EDS. However, this measure alone does not provide insights into subject alertness (i.e. whether individuals alert and awake, or groggy and awake). To address this limitation, we analyzed scored microsleep events during the MWT, which revealed that participants receiving treatment with the orexin agonist danavorexton not only had delayed onset to sustained sleep but also had fewer brief episodes of microsleep prior to sleep onset. Research is ongoing to better define microsleeps [28] and to understand which microsleep metrics are most important to characterize (e.g. number of microsleeps, microsleep latency [time to first microsleep], microsleep rate, and microsleep duration [29, 30]). As an example, Des Champs de Bioshebert et al. [31] compared microsleep latency with SOL in individuals treated for OSA or hypersomnia and found that although MSEs were seen in most individuals (with increased MSE in the afternoon), microsleep latency had a similar classification performance (measured by area under the curve) to traditional MWT SOL in identifying self-reported sleepiness. MSEs have also been shown to increase following sleep restriction in healthy young adults and to correlate with the KSS, thus providing a potential objective marker of daytime sleepiness [30]. Because manual scoring of microsleep is laborious and presents a barrier to routine quantification of microsleeps, several investigators are now exploring automated MSE scoring [32–34].

In our dataset, microsleep rate appears to be a sensitive treatment marker that provides additional information compared with MWT SOL. We considered but did not select several alternative microsleep metrics, discussed here. Because treatment could increase MWT SOL (and thus the duration of the MWT) by an order of magnitude, we felt the total count of microsleeps per session would be confounded with treatment effect (i.e. a count of two microsleeps has a very different interpretation in a 4-minute test vs. a 40-minute test). We also considered time to first microsleep as a possible metric, but judged it to be unreliable in our dataset, as several participants on treatment underwent a single early microsleep at approximately 5 minutes but then maintained wakefulness (with no additional microsleeps) for a full subsequent 40 minutes. Additionally, we believe that a single event is insufficient to describe an individual’s capacity to remain awake over a lengthy period of time. Furthermore, future work can explore Hurdle models (i.e. statistics conditional on appearance of microsleeps) of microsleep duration or other temporal metrics. In future studies, it would be interesting to determine whether a microsleep endpoint is more powerful than MWT score and whether MSEs offer better insight into response fluctuations over time (both across the four MWT sessions and over the treatment duration). In addition to microsleep analysis, we explored an artificial intelligence (AI)-derived metric (sleepiness score), which represents sleep probability as assessed using a deep learning model. We demonstrated that this metric correlates with the more widely established θ/α biomarker of sleepiness. Furthermore, we show that this biomarker is sensitive to treatment, presumably because it is less “noisy” than the θ/α ratio (Figure 2). Although this AI-based measure operates as a “black box” method, it offers potential robustness because it was able to evaluate features beyond θ/α and has been developed using a large dataset encompassing thousands of people.

Our findings show that sleep probability and the θ/α ratio are informative parameters for assessing increasing sleepiness during the MWT. Both participants who were treated and those who received placebo exhibited similar levels of initial sleepiness (similar intercepts). However, the rate of increase in sleepiness was significantly lower in the treated groups. We also observed a strong correlation between the rate of increase in log-sleepiness and inverse SOL for both participants with NT1 and NT2. From a technical point of view, this validates our use of a linear fit in this dataset. More fundamentally, it indicates that individuals with narcolepsy begin to experience increases in sleepiness immediately after being placed in a soporific situation. Potentially, the rate of increase in log-sleepiness could be measured outside the clinic and could provide an alternative measure to MWT SOL.

The siesta effect is believed to represent a particular period of vulnerability for which sleep debt has accumulated since the early morning, but the wake-promoting effect of the circadian clock has not yet manifested [35, 36]. This effect is difficult to observe in the untreated narcolepsy population due to the typical early MWT SOL throughout the day. In our study, danavorexton was infused at a constant rate throughout the day, resulting in a ceiling effect (40 minutes) for the morning MWT sessions. Interestingly, this strong treatment effect revealed a siesta effect in our study population, similar to the MWT/Multiple Sleep Latency Test (MSLT) feature found in normal untreated participants [32]. This phenomenon helped to clarify potential biomarkers that may be capable of predicting sleep onset by leveraging the additional MWT metrics reported herein.

An important limitation of our study is that it does not contain a normative group, or indeed any non-narcoleptic individuals. We anticipate that healthy individuals may be able to maintain a high level of alertness during the MWT, at least initially, after which sleepiness may begin to increase. If so, the simple linear fit model that appears to be appropriate in our dataset may not apply. It will therefore be important to repeat this analysis in a dataset that contains healthy controls. Related limitations of our study are that the study size was relatively small and that data were acquired at a single site, so it remains to be seen how well results generalize to larger studies across multiple sites. In addition, we were not able to directly link microsleeps to our AI-derived sleepiness score measure because the 15-second timescale of AI predictions is larger than the defined microsleep durations (which here we defined as a range of 3 to <15 seconds). Recent work has highlighted the possibility of sleep scoring on much smaller timescales [37], which would allow direct comparison of these metrics. A final limitation of this work is that we were not able to explore correlations to objective vigilance tests (e.g. the Psychomotor Vigilance Test [38]) or the Sustained Attention Reaction Test (SART [39]), which assess sustained attention (for example, by assessing microsleeps during SART). These will be additional correlations of interest that should be addressed in future studies.

Our study highlights the importance of considering MSEs in addition to SOL to give a richer description of EDS. We also propose new sleep probability metrics and the θ/α ratio as valuable indicators of increasing sleepiness during the MWT. These findings contribute to a better understanding of the effects of treatment on wakefulness and the mechanisms underlying sleep onset. The endpoints explored here may be of value in other protocols, for example, the MSLT [40] or the evaluation of EDS during 24- to 48-hour EEG recordings as performed in some centers for the evaluation of hypersomnia [41–43]. Our long-term goal is to identify endpoints for monitoring EDS that could be applied in settings outside the MWT, ideally leveraging emerging technologies for at-home EEG monitoring [44, 45].

## Supplementary material

Supplementary material is available at *SLEEP* online.

zsae148_suppl_Supplementary_Material

## Data Availability

The data that support the findings of this study are available from the corresponding author upon reasonable request.
