# Supplementary material for: Novel biomarkers derived from the Maintenance of Wakefulness Test as predictors of sleepiness and response to treatment
Source: Sleep. 2024 Jul 2;47(12):zsae148. doi: 10.1093/sleep/zsae148 (PMC11632192; doi:10.1093/sleep/zsae148)
Supplement: zsae148_suppl_Supplementary_Material [file zsae148_suppl_supplementary_material.docx]

# Supplementary Material:

**Novel biomarkers derived from the Maintenance of Wakefulness Test as predictors of sleepiness and response to treatment**

Brian Tracey^1^, Mark Culp^2^, Stephan Fabregas^3^, Emmanuel Mignot^4^, Derek L. Buhl^1,†,‡^ and Dmitri Volfson^1,^*^,†^

^1^Takeda Development Center Americas, Inc., Lexington, MA, USA

^2^Stat Tenacity, LLC, Saline, MI, USA

^3^Signal Insights, LLC, Cambridge, MA, USA

^4^Stanford Department of Psychiatry and Behavioral Sciences, Center for Sleep Sciences and Medicine, Stanford University Medical School, Palo Alto, CA, USA

*Corresponding author. Dmitri Volfson, Takeda Development Center Americas, Inc., 95 Hayden Avenue, Lexington, MA, 02421-7942, USA. Email: [Dmitri.volfson@takeda.com](mailto:Dmitri.volfson@takeda.com).

^†^Co-senior authors.

^‡^At the time the work was performed.

**Table of contents**

**Supplementary Figure S1:** Example waveform display used by sleep scorers.

**Supplementary Table S1.** Linear Mixed-Model Results for Participants with NT1, Baseline-Adjusted Changes in Sleepiness-Related Endpoints

**Supplementary Table S2.** Linear Mixed-Model Results for NT2 Cohorts, Baseline-Adjusted Changes in Sleepiness-Related Endpoints

**Supplementary Table S3.** Correlations Versus Inverse Maintenance of Wakefulness Test SOL and Between Sleepiness Score and θ/α metrics

**Supplementary Table S4.** Correlations Versus Maintenance of Wakefulness Test Microsleep (MSE) Parameters

**Supplementary Table S5.** Correlations Versus Karolinska Sleepiness Scale (KSS) Parameters


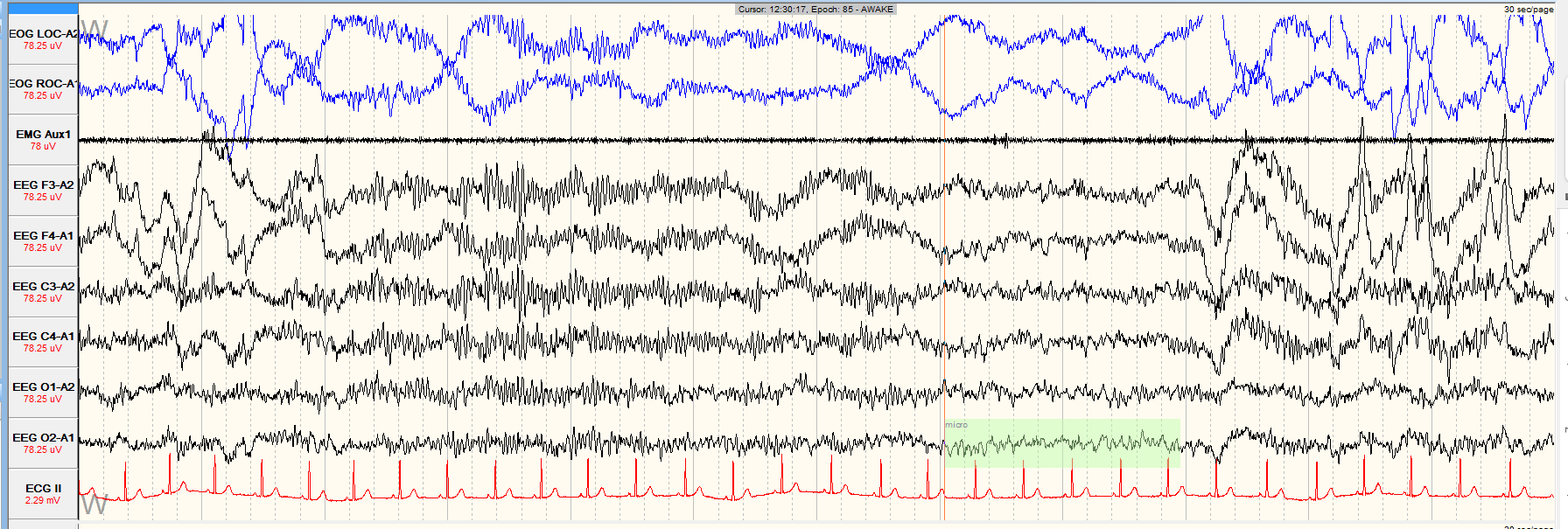


**Supplementary Figure S1:** Example waveform display used by sleep scorers.

The area shaded green (see EEG 02-A1, 2nd to bottom trace) is marked as a microsleep. Microsleeps were identified as a slowing in the EEG (shift from α to θ), often accompanied by slow rolling eye movements and occasionally accompanied by sleep spindles and/or K complexes.

**Supplementary Table S1.** Linear Mixed-Model Results for Participants with NT1, Baseline-Adjusted Changes in Sleepiness-Related Endpoints

| **Diagnosis** | **Endpoint** | **Contrast** | **Cohort** | **Estimate** | **SE** | ***p*-value** |
| --- | --- | --- | --- | --- | --- | --- |
| NT1 | SOL | day1 - (day-1) | Bp | –0.48 | 1.94 | 0.9449 |
| NT1 | SOL | day7 - (day-1) | Bp | –0.13 | 1.94 | 0.9942 |
| NT1 | MSE rate | day1 - (day-1) | Bp | 0.011 | 0.45 | 0.9997 |
| NT1 | MSE rate | day7 - (day-1) | Bp | 0.001 | 0.42 | 1.0000 |
| NT1 | MSE duration | day1 - (day-1) | Bp | 0.791 | 0.36 | 0.0732 |
| NT1 | MSE duration | day7 - (day-1) | Bp | 0.045 | 0.34 | 0.9906 |
| NT1 | avg sleepiness score | day1 - (day-1) | Bp | 0.68 | 0.43 | 0.2087 |
| NT1 | avg sleepiness score | day7 - (day-1) | Bp | 0.25 | 0.43 | 0.7670 |
| NT1 | intercept, sleepiness score | day1 - (day-1) | Bp | 0.52 | 0.29 | 0.1362 |
| NT1 | intercept, sleepiness score | day7 - (day-1) | Bp | 0.27 | 0.29 | 0.5448 |
| NT1 | slope, sleepiness score | day1 - (day-1) | Bp | 0.06 | 0.20 | 0.9224 |
| NT1 | slope, sleepiness score | day7 - (day-1) | Bp | 0.16 | 0.20 | 0.6259 |
| NT1 | avg theta/alpha | day1 - (day-1) | Bp | 0.08 | 0.19 | 0.8535 |
| NT1 | avg theta/alpha | day7 - (day-1) | Bp | 0.29 | 0.19 | 0.2181 |
| NT1 | intercept theta/alpha | day1 - (day-1) | Bp | 0.17 | 0.10 | 0.1631 |
| NT1 | intercept theta/alpha | day7 - (day-1) | Bp | 0.19 | 0.10 | 0.1047 |
| NT1 | slope theta/alpha | day1 - (day-1) | Bp | –0.05 | 0.06 | 0.6262 |
| NT1 | slope theta/alpha | day7 - (day-1) | Bp | –0.01 | 0.06 | 0.9551 |
| NT1 | KSS | day1 - (day-1) | Bp | -0.35 | 0.37 | 0.5500 |
| NT1 | KSS | day1 - (day-1) | Bp | -0.94 | 0.35 | 0.0166 |
| NT1 | SOL | day1 - (day-1) | B1 | 35.42 | 1.94 | <0.0001 |
| NT1 | SOL | day7 - (day-1) | B1 | 16.94 | 1.96 | <0.0001 |
| NT1 | MSE rate | day1 - (day-1) | B1 | –2.858 | 0.54 | <0.0001 |
| NT1 | MSE rate | day7 - (day-1) | B1 | –1.917 | 0.57 | 0.0023 |
| NT1 | MSE duration | day1 - (day-1) | B1 | –2.391 | 0.42 | <0.0001 |
| NT1 | MSE duration | day7 - (day-1) | B1 | –1.96 | 0.47 | 0.0001 |
| NT1 | avg sleepiness score | day1 - (day-1) | B1 | –4.37 | 0.43 | <0.0001 |
| NT1 | avg sleepiness score | day7 - (day-1) | B1 | –3.60 | 0.44 | <0.0001 |
| NT1 | intercept, sleepiness score | day1 - (day-1) | B1 | –0.34 | 0.29 | 0.3976 |
| NT1 | intercept, sleepiness score | day7 - (day-1) | B1 | –0.60 | 0.30 | 0.0876 |
| NT1 | slope, sleepiness score | day1 - (day-1) | B1 | –1.03 | 0.20 | <0.0001 |
| NT1 | slope, sleepiness score | day7 - (day-1) | B1 | –0.72 | 0.20 | 0.0009 |
| NT1 | avg theta/alpha | day1 - (day-1) | B1 | –1.21 | 0.19 | <0.0001 |
| NT1 | avg theta/alpha | day7 - (day-1) | B1 | –0.90 | 0.19 | <0.0001 |
| NT1 | intercept theta/alpha | day1 - (day-1) | B1 | –0.29 | 0.10 | 0.0068 |
| NT1 | intercept theta/alpha | day7 - (day-1) | B1 | –0.35 | 0.10 | 0.0013 |
| NT1 | slope theta/alpha | day1 - (day-1) | B1 | –0.22 | 0.06 | 0.0004 |
| NT1 | slope theta/alpha | day7 - (day-1) | B1 | –0.17 | 0.06 | 0.0094 |
| NT1 | KSS | day1 - (day-1) | B1 | -3.36 | 0.36 | <0.0001 |
| NT1 | KSS | day1 - (day-1) | B1 | -3.27 | 0.36 | <0.0001 |
| NT1 | SOL | day1 - (day-1) | B2 | 35.59 | 1.75 | <0.0001 |
| NT1 | SOL | day7 - (day-1) | B2 | 35.62 | 1.74 | <0.0001 |
| NT1 | MSE rate | day1 - (day-1) | B2 | –7.542 | 1.27 | <0.0001 |
| NT1 | MSE rate | day7 - (day-1) | B2 | –4.461 | 0.57 | <0.0001 |
| NT1 | MSE duration | day1 - (day-1) | B2 | –4.734 | 0.45 | <0.0001 |
| NT1 | MSE duration | day7 - (day-1) | B2 | –4.276 | 0.43 | <0.0001 |
| NT1 | avg sleepiness score | day1 - (day-1) | B2 | –4.16 | 0.39 | <0.0001 |
| NT1 | avg sleepiness score | day7 - (day-1) | B2 | –4.55 | 0.38 | <0.0001 |
| NT1 | intercept, sleepiness score | day1 - (day-1) | B2 | –0.09 | 0.26 | 0.9134 |
| NT1 | intercept, sleepiness score | day7 - (day-1) | B2 | –0.20 | 0.26 | 0.6586 |
| NT1 | slope, sleepiness score | day1 - (day-1) | B2 | –0.54 | 0.18 | 0.0056 |
| NT1 | slope, sleepiness score | day7 - (day-1) | B2 | –0.55 | 0.18 | 0.0046 |
| NT1 | avg theta/alpha | day1 - (day-1) | B2 | –0.44 | 0.17 | 0.0232 |
| NT1 | avg theta/alpha | day7 - (day-1) | B2 | –0.55 | 0.17 | 0.0028 |
| NT1 | intercept theta/alpha | day1 - (day-1) | B2 | 0.11 | 0.09 | 0.3950 |
| NT1 | intercept theta/alpha | day7 - (day-1) | B2 | 0.00 | 0.09 | 0.9981 |
| NT1 | slope theta/alpha | day1 - (day-1) | B2 | –0.09 | 0.05 | 0.1363 |
| NT1 | slope theta/alpha | day7 - (day-1) | B2 | –0.09 | 0.05 | 0.1320 |
| NT1 | KSS | day1 - (day-1) | B2 | -3.79 | 0.32 | <0.0001 |
| NT1 | KSS | day1 - (day-1) | B2 | -4.15 | 0.32 | <0.0001 |

Cohort values: Bp = NT1 placebo; B1 = NT1 11 mg danavorexton; B2 = NT1 44 mg danavorexton.

MSE, microsleep episode; NT1, narcolepsy type 1; SE, standard error; SOL, sleep onset latency.

**Supplementary Table S2.** Linear Mixed-Model Results for NT2 Cohorts, Baseline-Adjusted Changes in Sleepiness-Related Endpoints

| **Diagnosis** | **Endpoint** | **Contrast** | **Cohort** | **Estimate** | **SE** | ***p*-value** |
| --- | --- | --- | --- | --- | --- | --- |
| NT2 | SOL | day1 - (day-1) | Cp | 2.57 | 2.39 | 0.4585 |
| NT2 | SOL | day7 - (day-1) | Cp | 2.35 | 2.39 | 0.5169 |
| NT2 | MSE rate | day1 - (day-1) | Cp | –1.606 | 0.50 | 0.0039 |
| NT2 | MSE rate | day7 - (day-1) | Cp | –0.766 | 0.46 | 0.2182 |
| NT2 | MSE duration | day1 - (day-1) | Cp | –2.14 | 0.48 | <0.0001 |
| NT2 | MSE duration | day7 - (day-1) | Cp | –1.013 | 0.46 | 0.0691 |
| NT2 | avg sleepiness score | day1 - (day-1) | Cp | –0.49 | 0.39 | 0.3428 |
| NT2 | avg sleepiness score | day7 - (day-1) | Cp | 0.05 | 0.39 | 0.9797 |
| NT2 | intercept, sleepiness score | day1 - (day-1) | Cp | –0.53 | 0.18 | 0.0084 |
| NT2 | intercept, sleepiness score | day7 - (day-1) | Cp | –0.16 | 0.18 | 0.5675 |
| NT2 | slope, sleepiness score | day1 - (day-1) | Cp | –0.40 | 0.14 | 0.0080 |
| NT2 | slope, sleepiness score | day7 - (day-1) | Cp | –0.15 | 0.14 | 0.4359 |
| NT2 | avg θ/α | day1 - (day-1) | Cp | –0.22 | 0.14 | 0.2342 |
| NT2 | avg θ/α | day7 - (day-1) | Cp | 0.18 | 0.14 | 0.3554 |
| NT2 | intercept θ/α | day1 - (day-1) | Cp | –0.08 | 0.07 | 0.4167 |
| NT2 | intercept θ/α | day7 - (day-1) | Cp | 0.01 | 0.07 | 0.9592 |
| NT2 | slope θ/α | day1 - (day-1) | Cp | –0.10 | 0.04 | 0.0183 |
| NT2 | slope θ/α | day7 - (day-1) | Cp | –0.07 | 0.04 | 0.1568 |
| NT2 | KSS | day1 - (day-1) | Cp | -0.47 | 0.34 | 0.2896 |
| NT2 | KSS | day1 - (day-1) | Cp | 0.19 | 0.35 | 0.7961 |
| NT2 | SOL | day1 - (day-1) | C1 | 20.54 | 2.78 | <0.0001 |
| NT2 | SOL | day7 - (day-1) | C1 | 21.98 | 2.78 | <0.0001 |
| NT2 | MSE rate | day1 - (day-1) | C1 | –2.415 | 0.54 | <0.0001 |
| NT2 | MSE rate | day7 - (day-1) | C1 | –2.909 | 0.54 | <0.0001 |
| NT2 | MSE duration | day1 - (day-1) | C1 | –1.207 | 0.43 | 0.0136 |
| NT2 | MSE duration | day7 - (day-1) | C1 | –2.73 | 0.49 | <0.0001 |
| NT2 | avg sleepiness score | day1 - (day-1) | C1 | –3.13 | 0.45 | <0.0001 |
| NT2 | avg sleepiness score | day7 - (day-1) | C1 | –2.84 | 0.45 | <0.0001 |
| NT2 | intercept, sleepiness score | day1 - (day-1) | C1 | –0.06 | 0.21 | 0.9378 |
| NT2 | intercept, sleepiness score | day7 - (day-1) | C1 | –0.20 | 0.21 | 0.5471 |
| NT2 | slope, sleepiness score | day1 - (day-1) | C1 | –0.39 | 0.16 | 0.0307 |
| NT2 | slope, sleepiness score | day7 - (day-1) | C1 | –0.37 | 0.16 | 0.0388 |
| NT2 | avg θ/α | day1 - (day-1) | C1 | –0.37 | 0.17 | 0.0559 |
| NT2 | avg θ/α | day7 - (day-1) | C1 | –0.29 | 0.17 | 0.1564 |
| NT2 | intercept θ/α | day1 - (day-1) | C1 | 0.04 | 0.08 | 0.8077 |
| NT2 | intercept θ/α | day7 - (day-1) | C1 | 0.02 | 0.08 | 0.9509 |
| NT2 | slope θ/α | day1 - (day-1) | C1 | –0.10 | 0.04 | 0.0456 |
| NT2 | slope θ/α | day7 - (day-1) | C1 | –0.10 | 0.04 | 0.0601 |
| NT2 | KSS | day1 - (day-1) | C1 | -1.63 | 0.40 | 0.0001 |
| NT2 | KSS | day1 - (day-1) | C1 | -2.00 | 0.40 | <0.0001 |
| NT2 | SOL | day1 - (day-1) | C2 | 30.99 | 2.47 | <0.0001 |
| NT2 | SOL | day7 - (day-1) | C2 | 28.27 | 2.39 | <0.0001 |
| NT2 | MSE rate | day1 - (day-1) | C2 | –4.283 | 0.58 | <0.0001 |
| NT2 | MSE rate | day7 - (day-1) | C2 | –4.589 | 0.66 | <0.0001 |
| NT2 | MSE duration | day1 - (day-1) | C2 | –4.358 | 0.54 | <0.0001 |
| NT2 | MSE duration | day7 - (day-1) | C2 | –4.239 | 0.53 | <0.0001 |
| NT2 | avg sleepiness score | day1 - (day-1) | C2 | –4.41 | 0.40 | <0.0001 |
| NT2 | avg sleepiness score | day7 - (day-1) | C2 | –4.23 | 0.39 | <0.0001 |
| NT2 | intercept, sleepiness score | day1 - (day-1) | C2 | 0.11 | 0.19 | 0.7647 |
| NT2 | intercept, sleepiness score | day7 - (day-1) | C2 | 0.06 | 0.18 | 0.9034 |
| NT2 | slope, sleepiness score | day1 - (day-1) | C2 | –0.58 | 0.14 | 0.0001 |
| NT2 | slope, sleepiness score | day7 - (day-1) | C2 | –0.44 | 0.14 | 0.0030 |
| NT2 | avg theta/alpha | day1 - (day-1) | C2 | –0.76 | 0.15 | <0.0001 |
| NT2 | avg theta/alpha | day7 - (day-1) | C2 | –0.87 | 0.14 | <0.0001 |
| NT2 | intercept theta/alpha | day1 - (day-1) | C2 | 0.07 | 0.07 | 0.4963 |
| NT2 | intercept theta/alpha | day7 - (day-1) | C2 | –0.10 | 0.07 | 0.2456 |
| NT2 | slope theta/alpha | day1 - (day-1) | C2 | –0.15 | 0.04 | 0.0004 |
| NT2 | slope theta/alpha | day7 - (day-1) | C2 | –0.11 | 0.04 | 0.0101 |
| NT2 | KSS | day1 - (day-1) | C2 | -2.01 | 0.35 | <0.0001 |
| NT2 | KSS | day1 - (day-1) | C2 | -1.25 | 0.34 | 0.0007 |

Cohort values: Cp = NT2 placebo; C1 = NT2 44 mg danavorexton; C2 = NT2 112 mg danavorexton.

MSE, microsleep episode; NT2, narcolepsy type 2; SE, standard error; SOL, sleep onset latency.

**Supplementary Table S3.** Correlations Versus Inverse Maintenance of Wakefulness Test SOL and Between Sleepiness Score and θ/α metrics

| Correlation (rmcorr) | Correlation, lower bound | Correlation, upper bound | p value | x | y | Diagnosis |
| --- | --- | --- | --- | --- | --- | --- |
| 0.896 | 0.850 | 0.928 | <0.0001 | 1/ SOL | sleepiness slope | NT1 |
| 0.451 | 0.284 | 0.592 | <0.0001 | 1/ SOL | sleepiness average | NT1 |
| -0.036 | -0.226 | 0.156 | 0.7132 | 1/ SOL | sleepiness intercept | NT1 |
| 0.830 | 0.759 | 0.882 | <0.0001 | 1/ SOL | theta/alpha slope | NT1 |
| 0.574 | 0.430 | 0.690 | <0.0001 | 1/ SOL | theta/alpha average | NT1 |
| 0.115 | -0.078 | 0.300 | 0.2408 | 1/ SOL | theta/alpha intercept | NT1 |
| 0.902 | 0.858 | 0.932 | <0.0001 | sleepiness slope | theta/alpha slope | NT1 |
| 0.261 | 0.073 | 0.431 | 0.0072 | 1/ SOL | MSE rate | NT1 |
| -0.263 | -0.574 | 0.115 | 0.1682 | 1/ SOL | MSE avg duration | NT1 |
| 0.950 | 0.931 | 0.964 | <0.0001 | 1/ SOL | sleepiness slope | NT2 |
| 0.504 | 0.367 | 0.620 | <0.0001 | 1/ SOL | sleepiness average | NT2 |
| -0.089 | -0.254 | 0.081 | 0.3055 | 1/ SOL | sleepiness intercept | NT2 |
| 0.429 | 0.280 | 0.557 | <0.0001 | 1/ SOL | MSE rate | NT2 |
| 0.098 | -0.205 | 0.383 | 0.5286 | 1/ SOL | MSE avg duration | NT2 |
| 0.844 | 0.787 | 0.887 | <0.0001 | 1/ SOL | theta/alpha slope | NT2 |
| 0.597 | 0.476 | 0.696 | <0.0001 | 1/ SOL | theta/alpha average | NT2 |
| -0.007 | -0.176 | 0.162 | 0.9371 | 1/ SOL | theta/alpha intercept | NT2 |
| 0.855 | 0.802 | 0.895 | <0.0001 | sleepiness slope | theta/alpha slope | NT2 |

y~x; x = independent variable; y = dependent variable; rmcorr = repeated measures correlation.

MSE, microsleep episode; NT1, narcolepsy type 1; NT2, narcolepsy type 2; SOL, sleep onset latency.

**Supplementary Table S4.** Correlations Versus Maintenance of Wakefulness Test Microsleep (MSE) Parameters

| Correlation (rmcorr) | Correlation, lower bound | Correlation, upper bound | p value | x | y | Diagnosis |
| --- | --- | --- | --- | --- | --- | --- |
| 0.279 | 0.092 | 0.447 | 0.0039 | MSE rate | sleepiness slope | NT1 |
| -0.002 | -0.193 | 0.190 | 0.9870 | MSE rate | sleepiness average | NT1 |
| -0.116 | -0.462 | 0.262 | 0.5505 | MSE avg duration | sleepiness slope | NT1 |
| 0.236 | 0.047 | 0.409 | 0.0153 | MSE rate | theta/alpha slope | NT1 |
| -0.058 | -0.247 | 0.136 | 0.5596 | MSE rate | theta/alpha average | NT1 |
| -0.183 | -0.515 | 0.196 | 0.3410 | MSE avg duration | theta/alpha slope | NT1 |
| 0.374 | 0.219 | 0.511 | <0.0001 | MSE rate | sleepiness slope | NT2 |
| 0.131 | -0.038 | 0.294 | 0.1287 | MSE rate | sleepiness average | NT2 |
| 0.099 | -0.204 | 0.385 | 0.5229 | MSE avg duration | sleepiness slope | NT2 |
| 0.250 | 0.084 | 0.402 | 0.0035 | MSE rate | theta/alpha slope | NT2 |
| 0.207 | 0.040 | 0.363 | 0.0159 | MSE rate | theta/alpha average | NT2 |
| 0.199 | -0.104 | 0.468 | 0.1960 | MSE avg duration | theta/alpha slope | NT2 |

y~x; x = independent variable; y = dependent variable; rmcorr = repeated measures correlation.

MSE, microsleep episode; NT1, narcolepsy type 1; NT2, narcolepsy type 2.

**Supplementary Table S5.** Correlations Versus Karolinska Sleepiness Scale (KSS) Parameters

| Correlation (rmcorr) | Correlation, lower bound | Correlation, upper bound | p value | x | y | Diagnosis |
| --- | --- | --- | --- | --- | --- | --- |
| 0.628 | 0.497 | 0.732 | <0.0001 | KSS | sleepiness slope | NT1 |
| -0.229 | -0.403 | -0.039 | 0.0190 | KSS | sleepiness intercept | NT1 |
| 0.281 | 0.095 | 0.449 | 0.0037 | KSS | sleepiness average | NT1 |
| 0.570 | 0.424 | 0.686 | <0.0001 | KSS | theta/alpha slope | NT1 |
| -0.161 | -0.342 | 0.032 | 0.1009 | KSS | theta/alpha intercept | NT1 |
| 0.329 | 0.146 | 0.490 | 0.0006 | KSS | theta/alpha average | NT1 |
| 0.635 | 0.504 | 0.737 | <0.0001 | KSS | 1 / SOL | NT1 |
| 0.335 | 0.154 | 0.495 | 0.0005 | KSS | MSE rate | NT1 |
| -0.036 | -0.397 | 0.335 | 0.8533 | KSS | MSE avg duration | NT1 |
| 0.357 | 0.200 | 0.496 | <0.0001 | KSS | sleepiness slope | NT2 |
| 0.031 | -0.139 | 0.199 | 0.7231 | KSS | sleepiness intercept | NT2 |
| 0.294 | 0.131 | 0.441 | 0.0005 | KSS | sleepiness average | NT2 |
| 0.322 | 0.162 | 0.466 | 0.0001 | KSS | theta/alpha slope | NT2 |
| -0.066 | -0.232 | 0.104 | 0.4483 | KSS | theta/alpha intercept | NT2 |
| 0.321 | 0.161 | 0.465 | 0.0001 | KSS | theta/alpha average | NT2 |
| 0.416 | 0.265 | 0.546 | <0.0001 | KSS | 1 / SOL | NT2 |
| 0.207 | 0.039 | 0.363 | 0.0161 | KSS | MSE rate | NT2 |
| -0.082 | -0.370 | 0.220 | 0.5975 | KSS | MSE avg duration | NT2 |

y~x; x = independent variable; y = dependent variable; rmcorr=repeated measures correlation.

MSE, microsleep episode; NT1, narcolepsy type 1; NT2, narcolepsy type 2.
